# OsCAldOMT1 is a bifunctional *O*-methyltransferase involved in the biosynthesis of tricin-lignins in rice cell walls

**DOI:** 10.1038/s41598-019-47957-0

**Published:** 2019-08-12

**Authors:** Pui Ying Lam, Yuki Tobimatsu, Naoyuki Matsumoto, Shiro Suzuki, Wu Lan, Yuri Takeda, Masaomi Yamamura, Masahiro Sakamoto, John Ralph, Clive Lo, Toshiaki Umezawa

**Affiliations:** 1 Research Institute for Sustainable Humanosphere, Kyoto University, Gokasho, Uji, Kyoto 611-0011 Japan; 2 School of Biological Sciences, The University of Hong Kong, Pokfulam, Hong Kong China; 3 U.S Department of Energy Great Lakes Bioenergy Research Center, University of Wisconsin-Madison, Madison, WI 53726 USA; 4 Graduate School of Agriculture, Kyoto University, Sakyo-ku, Kyoto 606-8502 Japan; 5 Research Unit for Development of Global Sustainability, Kyoto University, Gokasho, Uji, Kyoto 611-0011 Japan; 6 Present Address: École polytechnique Fédérale de Lausanne, EPFL, 1015 Lausanne, Switzerland

**Keywords:** Molecular engineering in plants, Secondary metabolism

## Abstract

Lignin is a phenylpropanoid polymer produced in the secondary cell walls of vascular plants. Although most eudicot and gymnosperm species generate lignins solely via polymerization of *p*-hydroxycinnamyl alcohols (monolignols), grasses additionally use a flavone, tricin, as a natural lignin monomer to generate tricin-incorporated lignin polymers in cell walls. We previously found that disruption of a rice *5-HYDROXYCONIFERALDEHYDE* O*-METHYLTRANSFERASE* (*OsCAldOMT1*) reduced extractable tricin-type metabolites in rice vegetative tissues. This same enzyme has also been implicated in the biosynthesis of sinapyl alcohol, a monolignol that constitutes syringyl lignin polymer units. Here, we further demonstrate through in-depth cell wall structural analyses that *OsCAldOMT1*-deficient rice plants produce altered lignins largely depleted in both syringyl and tricin units. We also show that recombinant OsCAldOMT1 displayed comparable substrate specificities towards both 5-hydroxyconiferaldehyde and selgin intermediates in the monolignol and tricin biosynthetic pathways, respectively. These data establish OsCAldOMT1 as a bifunctional *O*-methyltransferase predominantly involved in the two parallel metabolic pathways both dedicated to the biosynthesis of tricin-lignins in rice cell walls. Given that cell wall digestibility was greatly enhanced in the *OsCAldOMT1*-deficient rice plants, genetic manipulation of *CAldOMT*s conserved in grasses may serve as a potent strategy to improve biorefinery applications of grass biomass.

## Introduction

Grasses, including cereals classified in the major monocot family Poaceae, show great potential as a source of lignocellulosic biomass, which is primarily composed of secondary cell walls produced in vascular tissues. A large amount of lignocellulosic biomass is produced worldwide annually as agricultural residues from grass grain crops including maize, wheat, rice, barley and sorghum. In addition, the so-called grass biomass crops, such as *Miscanthus*, *Erianthus*, switchgrass, and bamboos, are attracting attention as new sources of biomass for the production of biofuels and biochemicals, especially because of their superior lignocellulosic productivity and processability when compared with those of typical softwood and hardwood species, currently used in the timber and pulp industries^1,2^. To further improve our capacity to manipulate grass biomass by molecular breeding approaches, it is becoming increasingly important to deepen our understanding of the formation, structure, and properties of secondary cell walls in grasses^2–4^.

Lignin is a complex phenylpropanoid polymer derived from oxidative coupling of *p*-hydroxycinnamyl alcohols, namely monolignols, and related compounds, and typically accounts for 15%–30% of lignocellulosic material. By encrusting cell wall polysaccharides, i.e., cellulose and hemicelluloses, lignin confers enhanced mechanical strength, imperviousness, and resistance to pathogens^5–7^. Although the polymer is vital for all vascular plants to maintain their essential functions of water transport and structural support, lignin has long been considered a major obstacle for polysaccharide-oriented biomass utilization, including chemical pulping and fermentable sugar production. Accordingly, lignin bioengineering studies have traditionally targeted plants with reduced lignin content and/or altered lignin chemical structure to mitigate such lignin recalcitrance in polysaccharide utilization processes^5–8^. More recently, as lignin has been increasingly viewed as a viable source for biomass-derived aromatic fuels and commodities, alternative bioengineering approaches that manipulate lignin content/structure to improve lignin-oriented biomass utilization strategies are also becoming an important research subject^2,9,10^.

**Figure 1:**
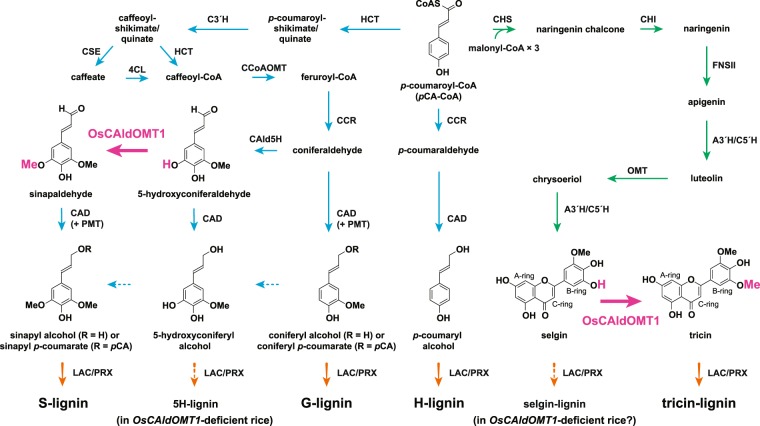
Proposed monolignol and tricin biosynthetic pathways in rice. HCT, *p*-hydroxycinnamoyl-CoA:quinate/shikimate *p*-hydroxycinnamoyltransferase; C3′H, *p*-coumaroyl ester 3-hydroxylase; CSE, caffeoyl shikimate esterase; 4CL: 4-coumaroyl-CoA ligase; cinnamoyl-CoA reductase; CCoAOMT, caffeoyl-CoA *O*-methyltransferase; CCR, cinnamoyl-CoA reductase; CAld5H, coniferaldehyde 5-hydroxylase; CAldOMT, 5-hydroxyconiferaldehyde *O*-methyltransferase; CAD, cinnamyl alcohol dehydrogenase; PMT, *p*-coumaroyl-CoA:monolignol transferase; CHS, chalcone synthase; CHI, chalcone isomerase; FNSII, flavone synthase II; A3′H/C5′H, apigenin 3′-hydroxylase/chrysoeriol 5′-hydroxylase; OMT, *O*-methyltransferase; LAC, laccase; PRX, peroxidase.

There is accumulating evidence that the structure and biosynthesis of lignins in grasses are distinctively different from those in gymnosperms (softwoods) and eudicots (including hardwoods)^11^. As a prime example, many grasses appear to use tricin, a 3′,5′-dimethoxyflavone, for cell wall lignification, which contrasts gymnosperm and eudicot species that typically use the canonical monolignols as sole lignin monomers^12–14^. Tricin, as a natural lignin monomer generated outside the monolignol biosynthetic pathway (Fig. 1), undergoes dehydrogenative co-polymerization with monolignols exclusively via 4′–*O*–β-type radical coupling upon cell wall lignification in grasses, as in the way lignification takes place solely with monolignols in typical gymnosperms and eudicots^13^. Such tricin-incorporated lignins, or tricin-lignins, have been shown to exist commonly in grasses and are also found in some non-grass monocots such as curaua, coconut, and orchids, as well as in the dicot alfalfa, albeit at very low levels^14^. Although it has long been recognized that monolignol-derived lignins and flavonoids generally localize and function differently in plants^5,15,16^, the discovery of tricin-lignins indicates a tight relationship between the two major classes of plant metabolite derived from the phenylpropanoid pathway; however, it remains largely unknown how such tricin-lignins are biosynthesized and function in grass cell walls. Moreover, how the existence of tricin-lignins affects the usability of grass biomass remains poorly understood.

Monolignols and tricin are both major downstream metabolites in the phenylpropanoid biosynthetic pathway in grasses. In general, after branching off from *p*-coumaroyl-CoA (*p*CA-CoA), a common precursor in the monolignol and flavonoid biosynthetic pathways, monolignols are synthesized through successive aromatic hydroxylations and *O*-methylations, along with side-chain reductions and occasionally with further side-chain acylations. In grasses, the major monolignol-type lignin monomers are *p*-coumaryl, coniferyl, and sinapyl alcohols, and their γ-*p*-coumaroylated derivatives, which together make up the canonical *p*-hydroxyphenyl (H), guaiacyl (G), and syringyl (S) units in lignin polymers, respectively, in cell walls (Fig. 1)^5,17^. On the other hand, the biosynthesis of tricin may involve the formation of the flavone backbone via the condensation of *p*CA-CoA with malonyl-CoA followed by chalcone isomerization and desaturation, leading to apigenin which is then converted into tricin through successive aromatic hydroxylations and *O*-methylations on the B-ring (Fig. 1). We recently identified some flavone biosynthetic enzymes responsible for the formation of tricin-lignins in rice cell walls by thorough characterization of rice mutant lines; rice mutants deficient in a rice *FLAVONE SYNTHASE II* (*OsFNSII* or *CYP93G1*)^18^ and a bifunctional *APIGENIN 3*′*-HYDROXYLASE*/*CHRYSOERIOL 5*′*-HYDROXYLASE* (*OsA3*′*H*/*C5*′*H* or *CYP75B4*)^19^ (Fig. 1) produced altered lignins completely devoid of tricin units and partially incorporating naringenin and apigenin, respectively, as a tricin surrogate, demonstrating predominant roles of these enzyme genes in tricin-lignin formation in rice cell walls. Both FNSII and A3′H/C5′H sequences appear to be highly conserved among grasses and likely function in the synthesis of tricin-lignins commonly present in grass cell walls^18,19^.

5-HYDROXYCONIFERALDEHYDE *O*-METHYLTRANSFERASE (CAldOMT or CAFFEIC ACID *O*-METHYLTRANSFERASE, COMT) is a key enzyme involved in the biosynthesis of S lignins in angiosperm species. In general, recombinant CAldOMTs accept many types of phenylpropanoid substrates but usually show notably higher catalytic efficiencies toward 5-hydroxyconiferaldehyde, a possible *in planta* substrate in the S lignin biosynthetic pathway that has diverged from the G lignin biosynthetic pathway^20–24^ (Fig. 1). In agreement with the proposed function of CAldOMT in S lignin biosynthesis, downregulation of *CAldOMT* typically results in reduced S lignin content often accompanied by the appearance of atypical 5-hydroxyguaiacyl (5H) lignin units via incorporation of the non-canonical 5-hydroxyconiferyl alcohol monomer upon lignification^23–29^ (Fig. 1). In some grasses, such as maize and sorghum, mutations in *CAldOMT* genes are associated with a brown midrib (bm) phenotype that often exhibits reduced lignin content and enhanced forage digestibility^30,31^. Similarly to such *bm* mutants, transgenic plants downregulated for *CAldOMT* typically display enhanced forage digestibility and/or biomass saccharification efficiency^23,26,31–38^. Hence, CAldOMT has been considered one of the promising targets in lignin bioengineering studies.

Previously, we demonstrated that a rice CAldOMT, OsCAldOMT1 (also known as OsCOMT1 or ROMT9), is requisite for S lignin biosynthesis in rice, a grass model species and a commercially important crop; an RNA-interference (RNAi)-derived *OsCAldOMT1*-knockdown rice (*OsCAldOMT1*-RNAi) was significantly depleted in S lignin units with increments in G and 5H lignin units^23^. Interestingly, however, in a separate work, we also found that a T-DNA insertional mutant rice deficient in the same gene (*OsCAldOMT1*-TDNA) was remarkably reduced in some extractable flavone metabolites including tricin and its *O*-conjugates in the vegetative tissues^39^. These observations collectively suggest that OsCAldOMT1 may have a dual role along both the monolignol and tricin biosynthetic pathways, which together direct the formation of tricin-lignins in rice cell walls; however, the precise involvement of OsCAldOMT1 in tricin-lignin biosynthesis in rice has not been completely addressed.

In this study, we provide evidence for the bifunctional role of OsCAldOMT1 in the formation of tricin-lignins in rice through in-depth cell wall structural analysis using histochemical, chemical, and two-dimensional (2D) NMR methods on the *OsCAldOMT1*-deficient rice lines. We also performed comparative kinetic assays of a recombinant OsCAldOMT1 using 5-hydroxyconiferaldehyde and selgin, potential *in planta* substrates of OsCAldOMT1. Our data firmly establish the bifunctional role of OsCAldOMT1 in generating both S and tricin lignin polymer units in rice cell walls. Taken together with the corroborative cell wall NMR data recently reported for *CAldOMT*-deficient maize^36^ and sorghum^40^, both of which showed reduced tricin-lignin contents in their cell walls, the bifunctional role of CAldOMT in tricin-lignin biosynthesis may be well conserved in the grass family.

## Results

### Sequence and expression analysis of *OsCAldOMT1*

We first performed a phylogenetic analysis to examine the phylogenetic relationship between OsCAldOMT1 and other CAldOMT proteins implicated in lignification. As shown in Supplementary Fig. S1, OsCAldOMT1 was clustered with other previously characterized grass CAldOMTs, including those in *Brachypodium distachyon*^35^, maize^36^, barley^38^, switchgrass^33^, wheat^41^, sugarcane^34^ and sorghum^40^ with high sequence identities (79%–83% identity) and separated from dicot CAldOMTs (47%–61% identity) in Arabidopsis^24,42–44^, poplar species^20,27^, *Medicago sativa*^26^, and *Nicotiana tabacum*^45^, all of which have been established as *bona fide* CAldOMTs functioning in S lignin biosynthesis (Supplementary Fig. S1).

Next, we examined the spatio-temporal expression pattern of *OsCAldOMT1* in wild-type rice (cv. Nipponbare) along with the other known/putative monolignol and tricin biosynthetic genes using the RiceXPro gene expression database^46^. *OsCAldOMT1* is concurrently expressed with monolignol biosynthetic genes, such as p*-COUMAROYL ESTER 3-HYDROXYLASE* (*OsC3*′*H1*)^47^, *CONIFERALDEHYDE 5-HYDROXYLASE* (*OsCAld5H1*)^48,49^, *CINNAMYL ALCOHOL DEHYDROGENASE* (*OsCAD2*)^50^ and p*-COUMAROYL-COA:MONOLIGNOL TRANSFERASE* (*OsPMT1*)^51^ (Fig. 1), with high expression levels in tissues in which lignification typically occurs (Supplementary Fig. S2). In addition, *OsCAldOMT1* shares a similar expression pattern with the known/putative tricin biosynthetic genes, including *CHALCONE SYNTHASE* (*OsCHS1*)^52^, *CHALCONE ISOMERASE* (*OsCHI*)^52,53^, *FLAVONE SYNTHASE II* (*OsFNSII*)^18,54^ and *APIGENIN 3*′*-HYDROXYLASE*/*CHRYSOERIOL 5*′*-HYDROXYLASE* (*OsA3*′*H/C5*′*H*)^39^ (Supplementary Fig. S2); among which *OsFNSII*^18^ and *OsA3*′*H/C5*′*H*^19^ have been shown to be indispensable for the formation of tricin-lignins in rice cell walls. These data are in line with our hypothesis that OsCAldOMT1 is involved in both the monolignol and tricin biosynthetic pathways in rice.

### Enzyme assay of recombinant OsCAldOMT1

**Figure 2:**
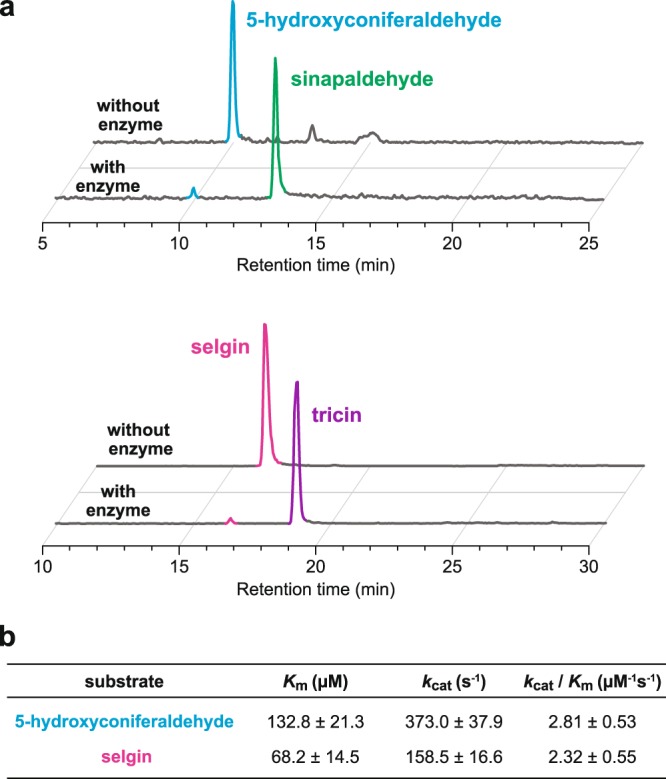
Kinetic assays for recombinant OsCAldOMT1. (**a**) HPLC chromatograms of reaction mixtures obtained for reactions of recombinant OsCAldOMT1 with 5-hydroxyconiferaldehyde (upper) and selgin (lower) substrates (detection by UV absorbance at 254 nm). (**b**) Kinetic parameters obtained for the reactions of recombinant OsCAldOMT1 with 5-hydroxyconiferaldehyde and selgin substrates. Values are means ± standard errors (*n* = 3).

In our previous biochemical study, recombinant OsCAldOMT1 was found to catalyze *O*-methylation of several possible intermediates in the monolignol biosynthetic pathway, such as caffeic acid, caffeoyl-CoA, 5-hydroxyferuloyl-CoA, caffealdehyde, 5-hydroxyconiferaldehyde, caffeyl alcohol, and 5-hydroxyconiferyl alcohol, among which 5-hydroxyconiferaldehyde, a key intermediate in S lignin biosynthesis (Fig. 1), was the most preferential substrate^23^. Here, we extend the enzyme assay on selgin, which is a logical substrate of CAldOMT in the tricin biosynthetic pathway (Fig. 1). Recombinant OsCAldOMT1^23^ produced tricin when incubated with selgin, demonstrating its capability to 5′-*O*-methylate selgin *in vitro* (Fig. 2a). Enzyme substrate specificity was then examined comparatively for 5-hydroxyconiferaldehyde and selgin. As a consequence, we found that the recombinant OsCAldOMT1 displayed comparable catalytic efficiencies (*k*_cat_/*K*_m_ > 2.3 µM^−1^s^−1^) towards both 5-hydroxyconiferaldehyde and selgin (Fig. 2b). Overall, our data further corroborate the dual functions of OsCAldOMT1 in tricin-lignin biosynthesis in rice.

### Phenotypes of *OsCAldOMT1*-deficient rice

To further investigate the *in planta* role of OsCAldOMT1, we re-examined our previously generated *OsCAldOMT1*-deficient rice lines, namely, the *OsCAldOMT1*-RNAi knockdown (cv. Nipponbare)^23^ and *OsCAldOMT1*-TDNA knockout (cv. Hwayoung)^39^ lines. We previously determined that *OsCAldOMT1* transcript levels were largely reduced down to ~1% of the wild-type levels in major vegetative tissues of *OsCAldOMT1*-RNAi^23^, whereas we expected a complete loss of functional OsCAldOMT1 activity in *OsCAldOMT1*-TDNA, which harbors a T-DNA insertion in the first exon of the *OsCAldOMT1* locus^39^. Both *OsCAldOMT1*-RNAi and *OsCAldOMT1*-TDNA plants were grown side-by-side with their wild-type controls for phenotypic characterization (Supplementary Fig. S3; Table 1). Under the present growth conditions, both *OsCAldOMT1*-deficient lines did not show significant differences in their growth parameters when compared with those of the wild-type controls, except that slight reductions in plant height were observed for *OsCAldOMT1*-TDNA (Supplementary Fig. S3; Table 1). The notable growth differences between *OsCAldOMT1*-RNAi and *OsCAldOMT1*-TDNA and between their corresponding wild-type plants, i.e., between WT1 and WT2 (Table 1), were probably because of their different genetic backgrounds.

**Table 1:** Growth properties of *OsCAldOMT1*-RNAi, *OsCAldOMT1*-TDNA and their wild-type controls (WT1 and WT2).

	WT1 (cv. Nipponbare)	*OsCAldOMT1*-RNAi (cv. Nipponbare)	WT2 (cv. Hwayoung)	*OsCAldOMT1*-TDNA (cv. Hwayoung)
plant height (cm)^*a*^	109.6 ± 8.4	116.3 ± 6.5	93.8 ± 3.5	88.5 ± 2.3*
culm length (cm)^*b*^	77.3 ± 4.5	73.1 ± 2.0	62.1 ± 3.0	59.9 ± 3.5
ear length (cm)	19.5 ± 2.0	17.3 ± 1.8	20.1 ± 3.7	18.5 ± 2.7
tiller number	8.0 ± 1.2	7.0 ± 0.7	11.0 ± 2.6	12.0 ± 2.8
ear number	8.0 ± 1.2	7.0 ± 0.7	13.2 ± 4.3	12.2 ± 2.5

Values refer to mean ± standard deviation. Asterisks indicate significant differences between transgenic and wild type (*n* = 5, Student’s *t*-test, *P < 0.05). ^*a*^Length between the base of aerial part and the tip of top leaf. ^*b*^Length between the base of aerial part and the base of panicle.

### Histochemical analysis of *OsCAldOMT1*-deficient rice cell walls

**Figure 3:**
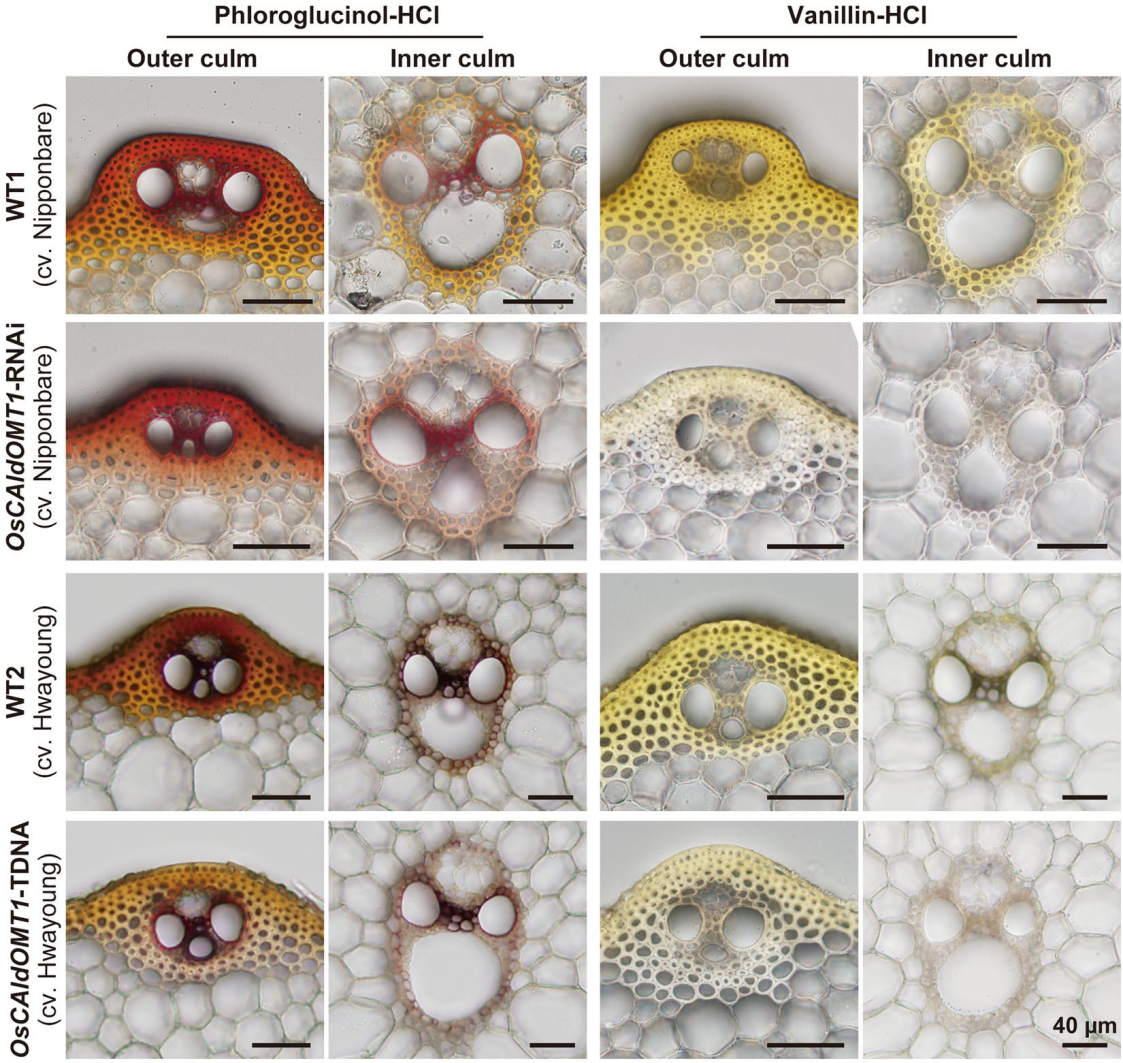
Histochemical analysis of rice culm tissues of *OsCAldOMT1*-RNAi, *OsCAldOMT1*-TDNA and their wild-type controls (WT1 and WT2). Transverse sections of culm tissues were subjected to phloroglucinol-HCl and vanillin-HCl stains for visualization of monolignol-derived lignins and cell-wall-bound flavonoids, respectively.

Transverse sections from developing culms of *OsCAldOMT1*-RNAi and *OsCAldOMT1*-TDNA plants at heading stage were subject to cell wall histochemical analysis using phloroglucinol-HCl and vanillin-HCl reagents, which stain monolignol-derived lignins and flavonoids, respectively^18^. As visualized by phloroglucinol-HCl, the cell wall anatomy and the distribution of lignified tissues in *OsCAldOMT1*-RNAi and *OsCAldOMT1*-TDNA culms were overall similar to those observed in the wild-type controls, although we observed slightly reduced lignin signals in the cortical sclerenchyma fibers in *OsCAldOMT1*-RNAi and *OsCAldOMT1*-TDNA culms compared to in the wild-type control culms (Fig. 3). In contrast, whereas the lignified cortical sclerenchyma fiber and vascular bundle cell walls of wild-type culms displayed intense and positive yellowish colorations upon vanillin-HCl flavonoid staining, the flavonoid signal was clearly depleted in both *OsCAldOMT1*-RNAi and *OsCAldOMT1*-TDNA culm cell walls, suggesting that cell-wall-bound flavonoid, presumably lignin-bound tricin, was substantially reduced by the disruption of *OsCAldOMT1* (Fig. 3).

### Lignocellulosic compositional analysis of *OsCAldOMT1*-deficient rice cell walls

The impacts of *OsCAldOMT1*-deficiency on the lignocellulosic composition in rice cell walls were further examined by a series of wet-chemical analyses. The Klason lignin analysis on the rice culm cell wall residue (CWR) samples revealed that lignin content in the *OsCAldOMT1*-RNAi and *OsCAldOMT1*-TDNA cell walls were reduced by 21% (from 142.4 to 112.6 mg/g CWR) and 30% (from 153.5 to 107.3 mg/g CWR) when compared with those of the wild-type controls, respectively (Table 2). In contrast, neutral-sugars analysis suggested that the amounts of arabinose and xylose released from hemicellulosic glycans were significantly increased in both *OsCAldOMT1*-RNAi and *OsCAldOMT1*-TDNA cell walls, whereas the amount of glucose released from crystalline cellulose remained constant; we also observed additional increments in amorphous glucans and galactans in the *OsCAldOMT1*-TDNA cell walls (Table 2). These data suggested that the lignin reductions in the *OsCAldOMT1*-deficient rice cell walls were at least partially compensated by relative increases in the levels of arabinoxylans.

**Table 2:** Cell wall chemical analysis and enzymatic saccharification efficiency data of *OsCAldOMT1*-RNAi, *OsCAldOMT1*-TDNA and their wild-type controls (WT1 and WT2).

	WT1 (cv. Nipponbare)	*OsCAldOMT1*-RNAi (cv. Nipponbare)	WT2 (cv. Hwayoung)	*OsCAldOMT1*-TDNA (cv. Hwayoung)
**Lignin content**				
Klason lignin (mg/g CWR)	142.4 ± 1.5	112.6 ± 3.2**	153.5 ± 1.4	107.3 ± 0.6**
**Lignin composition by thioacidolysis**				
S_thio_ (mol%)	46.1 ± 0.9	11.2 ± 1.5**	48.0 ± 0.3	15.3 ± 0.4**
G_thio_ (mol%)	48.4 ± 0.7	78.4 ± 3.0**	44.4 ± 0.3	75.7 ± 0.9**
H_thio_ (mol%)	5.5 ± 0.2	10.4 ± 1.5**	7.6 ± 0.3	9.0 ± 0.7*
5H_thio_ (peak area%)^*a*^	0.3 ± 0.0	2.0 ± 0.1**	0.3 ± 0.1	3.6 ± 0.1**
S_thio_/G_thio_	1.0 ± 0.0	0.1 ± 0.0**	1.1 ± 0.0	0.2 ± 0.0**
**Lignin composition by DFRC**				
S_DFRC-*p*CA_ (mol%)	17.8 ± 1.7	7.7 ± 0.3**	14.6 ± 0.5	9.0 ± 0.1**
S_DFRC-OH_ (mol%)	19.6 ± 0.3	9.0 ± 0.2**	14.2 ± 0.1	8.3 ± 0.1**
G_DFRC-*p*CA_ (mol%)	3.8 ± 0.3	5.1 ± 0.2**	4.0 ± 0.2	5.9 ± 0.1**
G_DFRC-OH_ (mol%)	51.2 ± 1.6	70.8 ± 1.6**	54.1 ± 0.6	67.3 ± 0.3**
H_DFRC-OH_ (mol%)	7.6 ± 0.4	7.4 ± 1.0	13.1 ± 0.3	9.5 ± 0.2**
S_DFRC-total_/G_DFRC-total_^*b*^	0.7 ± 0.0	0.2 ± 0.0**	0.5 ± 0.0	0.2 ± 0.0**
**Cell wall polysaccharides**				
Crystalline glucan (mg/g CWR)	456.1 ± 19.7	469.4 ± 4.4	380.2 ± 54.7	403.6 ± 20.1
Amorphous glucan (mg/g CWR)	30.9 ± 9.6	27.7 ± 11.6	49.2 ± 3.9	62.1 ± 3.6*
Arabinan (mg/g CWR)	32.3 ± 2.1	44.6 ± 2.0**	36.9 ± 3.6	52.6 ± 3.2**
Xylan (mg/g CWR)	130.8 ± 17.7	244.1 ± 39.2*	148.1 ± 20.3	242.1 ± 20.5**
Galactan (mg/g CWR)	12.6 ± 2.1	13.6 ± 2.9	15.2 ± 0.7	18.3 ± 1.2*
**Cell-wall-bound cinnamates**				
*p*CA (µmol/g CWR)	12.4 ± 0.5	5.5 ± 0.3**	10.9 ± 0.7	4.4 ± 0.3**
FA (µmol/g CWR)	4.1 ± 0.3	6.6 ± 0.1**	3.7 ± 0.2	5.2 ± 0.5**
**Cell wall saccharification efficiency**				
Glucose released after 6 h (mg/g CWR)^*c*^	172.9 ± 14.6	271.2 ± 22.1**	171.7 ± 8.2**	268.9 ± 13.1**
Glucose released after 24 h (mg/g CWR)^*c*^	179.1 ± 8.4	282.1 ± 1.8**	196.7 ± 10.8**	309.3 ± 5.8**

Values are means ± standard deviations. Asterisks indicate significant differences between transgenic and wild type (*n* = 3, Student’s *t*-test, *P < 0.05, **P < 0.01). ^*a*^Expressed as a percentage of the total peak area of S_thio_, G_thio_, H_thio_ and 5H_thio_. ^*b*^S_DFRC-total_ = S_DFRC-*p*CA_ + S_DFRC-OH_; G_DFRC-total_ = G_DFRC-*p*CA_ + G_DFRC-OH_. ^*c*^Expressed as glucose yield per destarched cell wall residue (CWR).

We also quantified cell-wall-bound *p*-coumarate (*p*CA) and ferulate (FA) as the corresponding free acids released via mild alkaline hydrolysis. Both the *OsCAldOMT1*-RNAi and *OsCAldOMT1*-TDNA cell walls were substantially depleted in *p*CA and conversely augmented in FA; approximately 56% (from 12.4 to 5.5 µmol/g CWR) and 59% (from 10.9 to 4.4 µmol/g CWR) reductions in *p*CA levels, and 62% (from 4.1 to 6.6 µmol/g CWR) and 39% (from 3.7 to 5.2 µmol/g CWR) increments in FA levels were observed for the *OsCAldOMT1*-RNAi and *OsCAldOMT1*-TDNA cell walls, respectively (Table 2). Given that, in typical grass cell walls, the majority of *p*CA is bound to lignins whereas (releasable) FA is mainly associated with hemicelluloses, in particular, arabinoxylans (Ralph, 2010), these changes in *p*CA and FA levels in the *OsCAldOMT1*-deficient cell walls can be attributed to relatively reduced levels of lignins and increases in arabinoxylans, respectively (Table 2).

### Lignin compositional analysis of *OsCAldOMT1*-deficient rice cell walls

We previously examined lignin composition in the *OsCAldOMT1*-RNAi^23^ using analytical thioacidolysis, which specifically cleaves β–*O*–4 linkages in lignin polymers and releases quantifiable monomeric compounds reflecting the polymer unit composition (Supplementary Fig. S4)^55^. Here, lignin composition in the *OsCAldOMT1*-RNAi and *OsCAldOMT1*-TDNA cell walls was further evaluated by thioacidolysis, as well as derivatization followed by reductive cleavage (DFRC) methods. As DFRC also cleaves β–*O*–4 linkages in lignin polymers, but, unlike thioacidolysis, retains the γ-acyl groups and releases quantifiable γ-*p*-coumaroylated monomeric compounds along with non-acylated (γ-free) monomeric compounds, it provides useful information about the γ-acylation status of lignin polymers in grasses (Supplementary Fig. S4)^56^.

Both thioacidolysis and DFRC suggested that S/G ratios were drastically reduced in the *OsCAldOMT1*-deficient rice cell walls when compared with the wild-type controls: thioacidolysis-derived S/G ratios (S_thio_/G_thio_) were reduced by 85% (from 1.0 to 0.1) and 81% (from 1.1 to 0.2) and DFRC-derived S/G ratios (S_DFRC-total_/G_DFRC-total_; S_DFRC-total_ = S_DFRC-OH_ + S_DFRC-*p*CA_, G_DFRC-total_ = G_DFRC-OH_ + G_DFRC-*p*CA_) by 68% (from 0.7 to 0.2) and 52% (from 0.5 to 0.2) in the *OsCAldOMT1*-RNAi and *OsCAldOMT1*-TDNA cell walls, respectively (Table 2; Supplementary Fig. S4), affirming that CAldOMT1 plays a key role in the biosynthesis of S lignins in rice^23^. DFRC further determined that both the non-acylated S (S_DFRC-OH_) and γ-*p*-coumaroylated S (S_DFRC-*p*CA_) monomeric compounds were proportionally reduced over the non-acylated G (G_DFRC-OH_) and γ-*p*-coumaroylated G (G_DFRC-*p*CA_) monomeric compounds (Table 2, Supplementary Fig. S4). In addition, thioacidolysis determined significantly increased levels of 5-hydroxyguaiacyl (5H)-type monomeric compounds (5H_thio_) in both *OsCAldOMT1*-RNAi and *OsCAldOMT1*-TDNA cell walls (Table 2; Supplementary Fig. S4), demonstrating the incorporation of unusual 5H units in lignins produced in the *OsCAldOMT1*-deficient rice cell walls.

### 2D NMR analysis of *OsCAldOMT1*-deficient rice lignins

**Figure 4:**
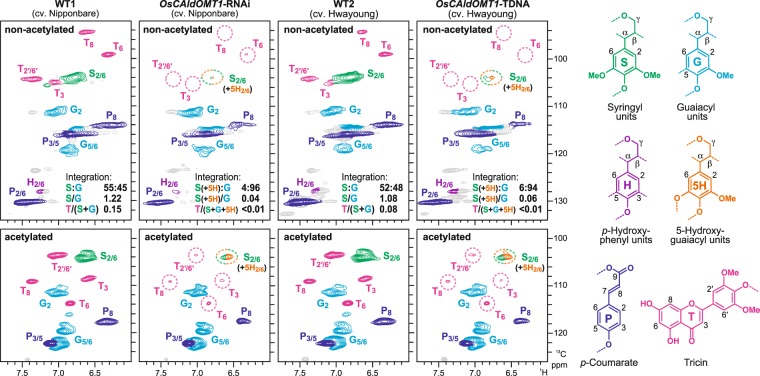
Aromatic sub-regions of short range ^1^H–^13^C correlation (HSQC) NMR spectra of non-acetylated (upper) and acetylated (lower) samples of lignin-enriched CWRs prepared from culm tissues of *OsCAldOMT1*-RNAi, *OsCAldOMT1*-TDNA and their wild-type controls (WT1 and WT2). Contour coloration matches that of the lignin substructure units shown. Volume-integration data for the major aromatic units are shown in the non-acetylated lignin spectra.

For more in-depth lignin structural analysis, we performed 2D HSQC NMR analysis on non-acetylated and acetylated lignin samples prepared from rice culm tissues. The aromatic regions of the HSQC NMR spectra collected for wild-type rice lignins displayed intense signals from the canonical S and G lignin units along with those from the grass-specific tricin and *p*CA units; these aromatic signals were predictably shifted after acetylation (Fig. 4). In line with the chemical analysis data (Table 2), our HSQC data demonstrated that S lignins are largely depleted in the *OsCAldOMT1*-deficient rice lignins. Volume integration of the HSQC signals roughly estimated the decrease of S/G ratios to be 97% (from 1.22 to 0.04) and 94% (from 1.08 to 0.06) in the non-acetylated *OsCAldOMT1*-RNAi and *OsCAldOMT1*-TDNA lignin spectra, respectively; S lignin signals in *OsCAldOMT1*-deficient lignin spectra could be overestimated because signals from newly appearing 5H lignin units considerably overlap with S lignin signals^27,28^ (Fig. 4). In addition, we observed sharp reductions of the tricin signals in the *OsCAldOMT1*-deficient rice lignin spectra. Compared with the wild-type control spectra, tricin signals were reduced by more than 98% in both non-acetylated *OsCAldOMT1*-RNAi and *OsCAldOMT1*-TDNA lignin spectra (Fig. 4). These data firmly established that OsCAldOMT1 is requisite for normal formation of tricin lignin units in addition to S lignin units in rice cell walls.

**Figure 5:**
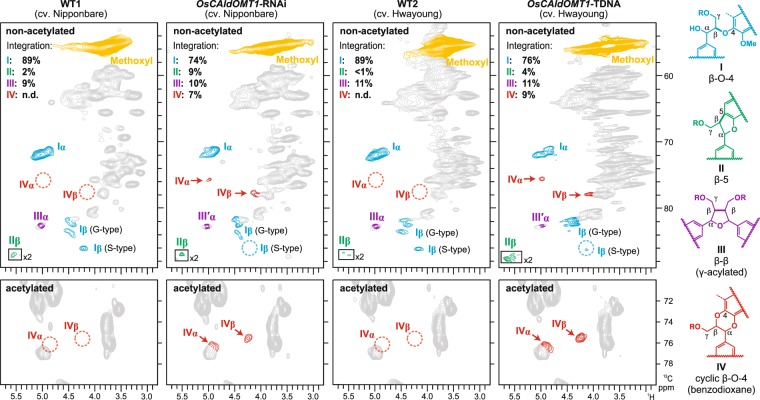
Aliphatic sub-regions of short range ^1^H–^13^C correlation (HSQC) NMR spectra of non-acetylated (upper) and acetylated (lower) samples of lignin-enriched CWRs prepared from culm tissues of *OsCAldOMT1*-RNAi, *OsCAldOMT1*-TDNA and their wild-type controls. Contour coloration matches that of the inter-monomeric linkages shown (WT1 and WT2). Volume integration data for the major units with their characteristic inter-monomeric linkages are shown in the non-acetylated lignin spectra.

The aliphatic side-chain regions of the HSQC spectra further provide information on the distribution of inter-monomeric linkages in the lignin polymers. Typical lignin linkage signals from β–*O*–4 (I), β–5 (II), and β–β (III) units were clearly seen in all wild-type and *OsCAldOMT1*-deficient lignin spectra (Fig. 5). Volume-integration analysis of these signals suggested that the proportions of β–*O*–4 units decreased, whereas the proportions of β–5 units increased in both *OsCAldOMT1*-RNAi and *OsCAldOMT1*-TDNA lignins (Fig. 5). In addition, new signals from the cyclic β–*O*–4 (benzodioxane) units (IV), which were not detected in the wild-type lignin spectra, were clearly visible in both the *OsCAldOMT1*-deficient rice lignin spectra [C_α_–H_α_ correlations from IV (IV_α_) at *δ*_C_/*δ*_H_ ~76/~5.0; C_β_–H_β_ correlations (IV_β_) from IV at *δ*_C_/*δ*_H_ ~78/~4.1], accounting for 7% and 9% of the total detected inter-monomeric linkage signals (I, II, III, and IV) in the spectra of non-acetylated *OsCAldOMT1*-RNAi and *OsCAldOMT1*-TDNA lignins, respectively (Fig. 5). After acetylation, the benzodioxane signals predictably shifted [C_α_–H_α_ correlations (IV_α_) at *δ*_C_/*δ*_H_ ~76/~4.9; C_β_–H_β_ correlations (IV_β_) at *δ*_C_/*δ*_H_ ~75/~4.2] (Fig. 5) and perfectly matched with the chemical shift data previously reported for several benzodioxane-containing lignin polymers^57–59^. This result indicates the incorporation of atypical catechol-type monomers such as 5-hydroxyconiferyl alcohol and/or selgin in lignification of the *OsCAldOMT1*-deficient rice lignins, as further discussed below (Supplementary Fig. S5).

### Enzymatic saccharification efficiency of *OsCAldOMT1*-deficient rice cell walls

We previously demonstrated that *OsCAldOMT1*-RNAi displayed enhanced biomass saccharification efficiency^23^. Here, we also evaluated the saccharification performance of *OsCAldOMT1*-TDNA together with *OsCAldOMT1*-RNAi under identical conditions. Consequently, we affirmed both the *OsCAldOMT1*-RNAi and *OsCAldOMT1*-TDNA cell walls displayed similarly enhanced enzymatic saccharification efficiency over their wild-type controls (Table 2); compared with the wild-type controls, the enhancement in glucose yields from the *OsCAldOMT1*-RNAi and *OsCAldOMT1*-TDNA cell walls after 24 h incubation was similarly around 60%.

## Discussion

In line with our previous study^23^, the two *OsCAldOMT1*-deficient rice lines analyzed in this study, i.e., *OsCAldOMT1*-RNAi and *OsCAldOMT1*-TDNA, both produced lignins largely depleted in S units and partially incorporated with atypical 5H units, as clearly demonstrated through in-depth cell wall structural analyses using chemical and NMR methods (Table 2, Figs 4 and 5). These results collectively support the notion that OsCAldOMT1 is a predominant CAldOMT functioning in S lignin biosynthesis in rice. Our analysis by DFRC further showed that both non-acylated and acylated S-type lignin degradation products (S_DFRC-OH_ and S_DFRC-*p*CA_) were proportionally reduced with relative increases in the corresponding G-type products (G_DFRC-OH_ and G_DFRC-*p*CA_) (Table 2, Supplementary Fig. S4). Recently, the existence of an alternative lignin biosynthetic pathway(s) that specifically leads to the production of grass-specific γ-*p*-coumaroylated lignin units has been suggested through the analysis of transgenic grass plants deficient in common lignin biosynthesis genes. For example, in rice, mutations of the *CAld5H* and *C3*′*H* genes, key regulators of the H/G/S lignin unit composition in eudicots^5–7^ (Fig. 1), predictably altered the H/G/S unit composition in the common non-acylated lignin units but exerted no detectable effects on the H/G/S unit composition in grass-specific γ-*p*-coumaroylated lignin units^47,49^. On the other hand, our current DFRC data suggest that OsCAldOMT1 may function in generating both non-acylated and γ-*p*-coumaroylated lignin units (Table 2, Supplementary Fig. S4).

Importantly, our histochemical and 2D NMR analysis firmly established that, along with S lignins, lignin-bound tricin was drastically reduced in the *OsCAldOMT1*-deficient rice cell walls (Figs 3 and 4), demonstrating the crucial role of OsCAldOMT1 in the biosynthesis of not only the sinapyl alcohol but also the tricin monomers for lignification; our NMR data suggested that lignin-integrated tricin units were nearly absent in the *OsCAldOMT1*-deficient rice cell walls (Fig. 4). Given that around 80% of tricin produced in typical grass straws can be lignin-bound^14^ and that we also observed substantial reductions of extractable tricin-*O*-conjugates in *OsCAldOMT1*-TDNA^39^, it is likely that OsCAldOMT1 plays a major role in the overall production of tricin-related metabolites in rice, including extractable tricin-*O*-conjugates and cell-wall-bound tricin-lignins.

Because lignin polymerization is a chemical process in which plants may incorporate any available phenolic compounds that can undergo phenoxy radical formation by laccases and/or peroxidases in lignifying cell walls, truncation of the monolignol biosynthetic pathway often results in the incorporation of pathway intermediates or their derivatives as non-canonical lignin monomers^8,60,61^; examples include the incorporation of caffeyl alcohol in a *CCoAOMT*-deficient plant^62^, ferulic acid in *CCR*-deficient plants^63^, *p*-hydroxycinnamaldehydes in *CAD*-deficient plants^50,57^ and, as we have demonstrated in this and previous studies with rice^23^, 5-hydroxyconiferyl alcohol in *CAldOMT*-deficient plants^25–27^. A similar phenomenon has also been observed when the tricin pathway is disrupted in grass species containing tricin-lignins; rice mutants deficient in *OsFNSII*^18^ and *OsA3*′*H/C5*′*H*^19^ produced lignins incorporating naringenin and apigenin, respectively, as non-canonical lignin monomers instead of the canonical tricin monomer.

In our *OsCAldOMT1*-deficient rice, the incorporation of 5-hydroxyconiferyl alcohol was evident by the appearance of the 5H-type monomeric compounds in the thioacidolysis-derived degradation products^23^ (Table 2, Supplementary Fig. S4). In addition, our 2D HSQC NMR analysis detected diagnostic signals from the benzodioxane linkages (Fig. 5), which can be uniquely derived from β–*O*–4-type radical coupling of a monolignol with 5-hydroxyguaiacyl end-groups (themselves derived from 5-hydroxyconiferyl alcohol incorporation) followed by internal trapping of quinone methide intermediates by the *o*-hydroxyl group^64^ (Supplementary Fig. S5). Such benzodioxanes, however, can be similarly derived from lignification of various types of *o*-diphenolic monomers^58,65–67^ and it is possible that analogous tricin pathway intermediates with *o*-diphenols such as selgin participate in lignification and produce benzodioxanes in *CAldOMT*-deficient grass lignins (Supplementary Fig. S5). This, however, is not likely for the *OsCAldOMT1*-deficient rice tested in this study because detection of any reasonable flavonoid signals failed, other than the small residual tricin signals in the HSQC NMR spectra of the *OsCAldOMT1*-deficient lignins (Fig. 4). Thus, it is likely that the benzodioxane units detected in the *OsCAldOMT1*-deficient lignins were mostly from the polymerization of 5-hydroxyconiferyl alcohol, rather than selgin derived from the tricin pathway (Fig. 1). The result was somewhat surprising because our previous metabolite analysis determined that selgin substantially over-accumulated in the vegetative tissues of *OsCAldOMT1*-TDNA^39^. It is currently unknown why selgin from the tricin pathway is not incorporated into lignins, whereas 5-hydroxyconiferyl alcohol from the monolignol pathway is readily incorporated in our *OsCAldOMT1*-deficient rice.

Although this study established that OsCAldOMT1 has comparable *in vitro* catalytic abilities towards 5-hydroxyconiferaldehyde and selgin, the potential *in vivo* substrates in the monolignol and tricin biosynthetic pathways, respectively (Fig. 2), such dual catalytic activity of grass CAldOMTs to catalyze both monolignol and flavonoid substrates has been documented. Prior to this work, OsCAldOMT1 was shown to catalyze *O*-methylation of not only monolignol-associated substrates^23^ but also 3′-*O*-methylation of a wide range of flavonoids, such as eriodictyol, luteolin, dihydroquercetin, quercetin, and rhamnetin^68,69^, as well as sequential 3′/5′-*O*-methylation of tricetin and/or myricetin^39,70^ under *in vitro* conditions. Likewise, other grass CAldOMT homologs including those in wheat^71,72^, maize^73^, barley^73^, and sorghum^40^ (Supplementary Fig. S1), have been reported to display *in vitro* OMT activities toward various flavone substrates in addition to monolignol-associated substrates.

*In planta* evidence for the involvement of CAldOMTs in tricin-lignin biosynthesis has also been provided in other grass species; cell wall analytical data recently reported for maize *bm3*^36^ and sorghum *bm12*^40^ mutants harboring defects in their primary *CAldOMT* genes indicated that both S and tricin lignin units were concomitantly depleted in their lignins when compared to the wild-type controls, suggesting that, similarly to in rice, CAldOMT plays a key role in both the S lignin and tricin biosynthesis. Given also the conserved CAldOMT sequences (Supplementary Fig. S1) and the wide occurrence of tricin-lignins among grasses^14^, it is plausible that the utilization of bifunctional CAldOMT that generates tricin-lignins is a common feature of grasses and therefore modification of lignin and flavonoid content/composition in grass biomass may be achieved by manipulating a single *CAldOMT* target gene. In addition, as we have confirmed with rice (Table 2)^23^, *CAldOMT*-deficiency has been shown to positively impact the efficiency of biomass saccharification in many grass crops^31,33,35–38^. Collectively, CAldOMT holds promise as a potent bioengineering target for manipulating grass biomass structure and properties for improved biorefinery applications.

## Methods

### Bioinformatics

A phylogenetic tree was constructed by the neighbor-joining method using MEGA7^74^. Bootstrapping with 1,000 replications was performed. Microarray-based gene expression profiles were retrieved from the Rice Expression Profile Database^46^.

### CAldOMT enzyme assay

Expression of recombinant OsCAldOMT1 in *Escherichia coli* and subsequent purification of the enzyme were carried out as described previously^22,23^. For enzymatic assays, selgin was synthesized according to an analogous synthetic route previously described for tricin^13^ and synthetic protocols for 5-hydroxyconiferaldehyde are described in Sakakibara *et al*.^75^. The typical reaction mixture (200 μl) contained 149 μl of 50 mM Tris-HCl (pH 7.5), 8 μl of 5 mM *S*-adenosyl-l-methionine, 20 μl of 5-hydroxyconiferaldehyde or selgin substrate solution in DMSO and 5 μg (23 μl) of recombinant enzyme in 50 mM Tris-HCl (pH 7.5)^23^. Three independent reactions for 8 different substrate concentrations ranging from 10 to 200 µM were carried out. After incubation for 20 min, the reaction was terminated by adding 200 μl of 2 M HCl and 10 μl of 1 mg ml^−1^ 3,4,5-trimethoxycinnamic acid in DMSO as an internal standard. The reaction mixture was extracted with ethyl acetate, evaporated and re-dissolved in 50 μl of methanol. After filtration through a PVDF membrane with a 0.45 μm pore size (Ultrafree-MC-HV; Merck Millipore, Tullagreen, Co. Cork, Ireland), the reaction mixtures were analyzed by Shimadzu LCMS-2020 (Shimadzu, Kyoto, Japan) with the following conditions: injection volume, 3 μl; column, Gemini 5 μm C18 110 Å 150 × 2 mm (Phenomenex, USA); solvents, 0.1% (v/v) formate/water (solvent A) and 0.1% (v/v) formate/acetonitrile (solvent B); solvent gradient protocol, linear gradient of 90% solvent A and 10% solvent B to 50% solvent A and solvent B for 20 min; UV detection, at 254 nm; MS ionization, ESI (+/−); MS interface, 350 °C; MS desolvation line and heat block, 200 °C; MS nebulizer gas, N_2_ at 1.5 l min^−1^. Compound identification was carried out based on comparisons of retention time and MS fragmentation pattern with authentic standards. Calibration curves for quantification were constructed using authentic standards and 254 nm UV absorbance for peak detection and area calculation. The Michaelis-Menten curves for the determination of *V*_max_ and *K*_m_ values were obtained with the GraphPad Prism ver. 8.1.2 program (GraphPad Software Inc, San Diego, CA).

### Plant materials

*OsCAldOMT1*-RNAi plants (cv. Nipponbare; T_3_ generation) were derived from Koshiba *et al*.^23^. *OsCAldOMT1*-TDNA plants (cv. Hwayoung; accession number: PFG_2B-50240) were originally from the Crop Biotech Institute at Kyung Hee University^76^, and the homozygous mutant and near-isogenic wild-type lines were isolated as described previously^39^. Rice seeds were surface sterilized, germinated and grown in a phytotron with a photoperiod of 12 h light (~30 °C) and 12 h dark (~24 °C). Mature plants (~45 days after heading) were phenotypically characterized, harvested and dried at ~27 °C for 30 days prior to cell wall analysis^18,23^.

### Histochemistry

Fresh hand-cut specimens were excised from rice culms at the heading stage, fixed, sectioned and stained by phloroglucinol-HCl or vanillin-HCl as described previously^18^. The stained sections were visualized under an Olympus BX51 microscope (Olympus Optical, Tokyo, Japan).

### Cell wall sample preparations

Extractive-free cell wall residues (CWRs) for chemical analysis were prepared from culm samples of matured rice plants^77^. For NMR analysis, CWRs (~300 mg) were further ball-milled using the Planetary Micro Mill Pulverisette 7 (Fritsch Industrialist, Idar-Oberstein, Germany) equipped with ZrO_2_ vessels containing ZrO_2_ ball bearings (600 rpm, 12 cycles of 10 min ball-milling with 5 min intervals), followed by digestion using crude cellulases (Cellulysin; Calbiochem, La Jolla, CA, USA) to give lignin-enriched CWRs^59^. Aliquots of the lignin-enriched CWRs (~15 mg) were dissolved in DMSO-*d*_6_/pyridine-*d*_5_ (4:1, v/v) and subjected to 2D NMR analysis. In parallel, the lignin-enriched CWRs (~15 mg) were further acetylated in DMSO/*N*-methylimidazole/acetic anhydride (2:1:1, v/v/v)^59,78^ and dissolved in chloroform-*d* for additional 2D NMR data analysis.

### Chemical analyses

Klason lignin^79^, sugar analysis^18^, quantitation of cell-wall-bound *p*CA and FA released by mild-alkaline hydrolysis^80^, analytical thioacidolysis^77,81^, and DFRC analysis^47,82^ were performed in accordance with the methods described in the literature.

### 2D HSQC NMR analysis

NMR spectra were acquired on a Bruker Biospin Avance III 800 system (800 MHz, Bruker Biospin, Billerica, MA, USA) equipped with a cryogenically-cooled 5-mm TCI gradient probe. Adiabatic 2D HSQC NMR experiments were carried out using a standard Bruker implementation (hsqcetgpsp.3, Bruker Biospin, Billerica, MA, USA) with parameters described in the literature^62,83^, and data processing and analysis were carried out as described previously^18,59^. The central solvent peaks were used as internal references for chemical shift calibration (*δ*_C_/*δ*_H_: DMSO, 39.5/2.49 ppm; chloroform, 77.0/7.26 ppm). For volume integration of lignin signals, C_2_–H_2_ correlations from G units, C_2_–H_2_/C_6_–H_6_ correlations from S units and C_2′_–H_2′_/C_6′_–H_6′_ correlations from tricin units were used. Signals from S and tricin units were logically halved. The percentages in Fig. 4 were calculated based on G + S = 100. Volume integrations of lignin intermonomeric linkages were carried out using C_α_–H_α_ contours from I, II, III and IV units. Signals from III were logically halved. The percentages shown in Fig. 5 were expressed based on I + II + III + IV = 100.

### Enzymatic saccharification

Enzymatic saccharification was carried out essentially in accordance with Hattori *et al*.^84^ and Lam *et al*.^18^. Briefly, destarched CWRs were digested by a cocktail of commercial cellulolytic enzymes that contain Celluclast 1.5 L, Novozyme 188 and Ultraflo L (Novozymes, Bagsværd, Denmark) dissolved in 50 mM sodium citrate (pH 4.8). After 6 and 24 h of cell wall digestion, the glucose yield was measured using Glucose C-II test kit (Wako Pure Chemical Industries, Osaka, Japan), according to the manufacturer’s instruction.

### Accession numbers

Sequence data of rice OsCAldOMT1 can be found in the EMBL/GenBank data libraries under accession number Q6ZD89. Accession numbers for the sequences used in the phylogenetic analysis (Supplementary Fig. S1) and *in silico* gene expression analysis (Supplementary Fig. S2) are listed in Supplementary Information.

## Supplementary information

Supplementary Info

## Data Availability

All data necessary to evaluate the conclusions in this study are included in the published paper and its Supplementary Information file. Additional data, if required, will be made available by the corresponding authors upon request.
