# Supplementary material for: OsCAldOMT1 is a bifunctional O-methyltransferase involved in the biosynthesis of tricin-lignins in rice cell walls
Source: Sci Rep. 2019 Aug 12;9:11597. doi: 10.1038/s41598-019-47957-0 (PMC6690965; doi:10.1038/s41598-019-47957-0)
Supplement: Supplementary file 1 — Supplementary Info [file 41598_2019_47957_MOESM1_ESM.pdf]

SUPPLEMENTARY INFORMATION

OsCAldOMT1 is a bifunctional *O*-methyltransferase involved in the biosynthesis of tricin-lignins in rice cell walls

Pui Ying Lam[1,2], Yuki Tobimatsu[1*], Naoyuki Matsumoto[1], Shiro Suzuki[1], Wu Lan[3,a], Yuri Takeda[1], Masaomi Yamamura[1], Masahiro Sakamoto[4], John Ralph[3], Clive Lo[2] and Toshiaki Umezawa[1,5*]

[1]Research Institute for Sustainable Humanosphere, Kyoto University, Gokasho, Uji, Kyoto 611-0011, Japan; [2]School of Biological Sciences, The University of Hong Kong, Pokfulam, Hong Kong, China; [3]U.S Department of Energy Great Lakes Bioenergy Research Center, University of Wisconsin-Madison, Madison, WI 53726, USA; [4]Graduate School of Agriculture, Kyoto University, Sakyo-ku, Kyoto 606-8502, Japan; [5]Research Unit for Development of Global Sustainability, Kyoto University, Gokasho, Uji, Kyoto 611-0011, Japan; Present address: [a]École polytechnique Fédérale de Lausanne, EPFL, 1015, Lausanne, Switzerland;

[*]Correspondence: Yuki Tobimatsu (ytobimatsu@rish.kyoto-u.ac.jp; Tel: +81-774-38-3626; Fax: +81-774-38-3682); Toshiaki Umezawa (tumezawa@rish.kyoto-u.ac.jp; Tel: +81-774-38-3625; Fax: +81-774-38-3682)

List of Materials

Figure S1. Phylogenetic analysis of CAldOMT proteins.

Figure S2. *In silico* gene expression analysis of wild-type rice.

Figure S3. Morphological phenotype of rice plants.

Figure S4. Lignin-derived products released by thioacidolysis and DFRC.

Figure S5. Generation of benzodioxanes in *OsCAldOMT1*-deficient rice lignin.

Supplementary References

(Figure S1)

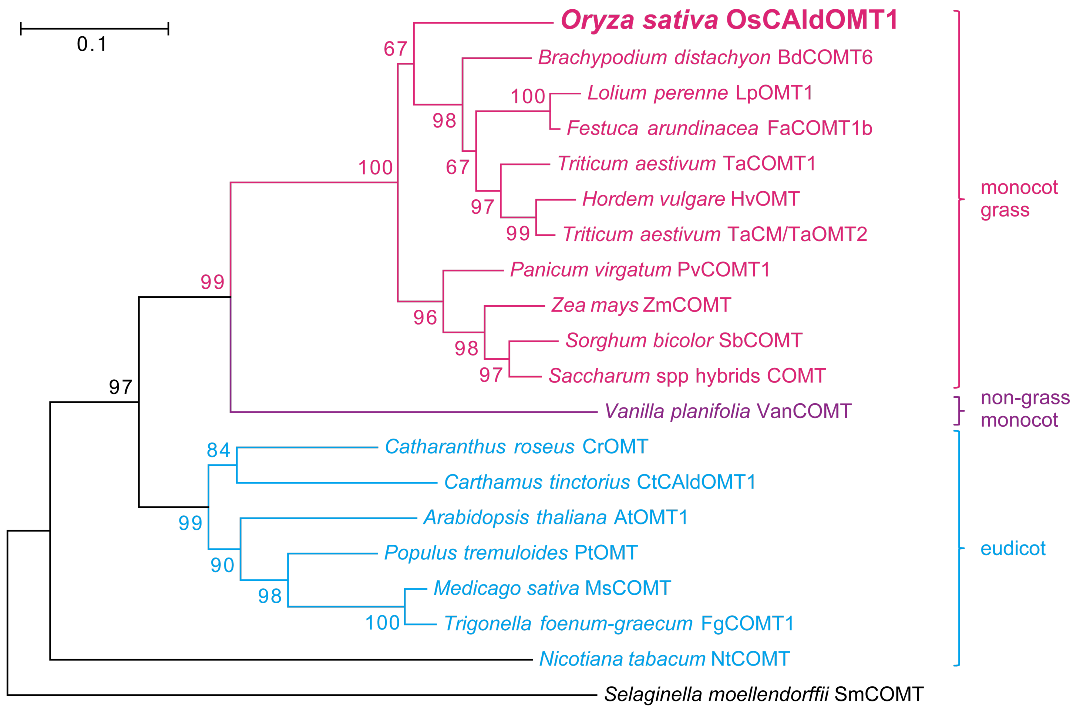

Figure S1. Phylogenetic analysis of CAldOMT proteins. A phylogenetic tree with 1,000 replicates of bootstrapping was constructed using the neighbor-joining method. Scale bar represents 0.1 amino acid substitutions per site. *Selaginella mollendorffii* SmCOMT was used as an outgroup.

In general, grass CAldOMTs (in magenta) cluster together and are sisters to those in eudicot (in blue) CAldOMT clades. Accession numbers for the sequences used in the phylogenetic analysis can be found in the EMBL/GenBank data libraries under accession numbers: Q6ZD89 (*Oryza sativa* OsCAldOMT1) (Koshiba *et al.*, 2013a), AAB03364 (*Zea mays* ZmCOMT) (Fornalé *et al.*, 2016), AAO43609 (*Sorghum bicolor* SbCOMT) (Eudes *et al.*, 2017), XP_003573470 (*Brachypodium distachyon* BdCOMT6) (Ho-Yue-Kuang *et al.*, 2015), HQ645965 (*Panicum virgatum* PvCOMT1) (Fu *et al.*, 2011), Q38J50 (*Triticum aestivum* TaCM/TaOMT2) (Ma and Xu, 2008), AJ231133 (*Saccharum* spp hybrids COMT) (Jung *et al.*, 2012), EF586876 (*Hordem vulgare* HvOMT) (Daly *et al.*, 2018), AAP23942 (*Triticum aestivum* TaCOMT1) (Ma and Xu, 2008), NP_200227 (*Arabidopsis thaliana* AtOMT1) (Goujon *et al.*, 2003), AAB61731 (*Populus tremuloides* PtOMT) (Osakabe *et al.*, 1999), P28002 (*Medicago sativa* MsCOMT) (Guo *et al.*, 2001), AF484252 (*Nicotiana tabacum* NtCOMT) (Pinçon *et al.*, 2001), Q8W013 (*Catharanthus roseus* CrCOMT) (Nakatsubo *et al.*, 2007), AB430466 (*Carthamus tinctorius* CtCAldOMT1) (Nakatsubo *et al.*, 2014) and GQ166949 (*Selaginella moellendorffii* SmCOMT) (Weng *et al.*, 2011).

(Figure S2)

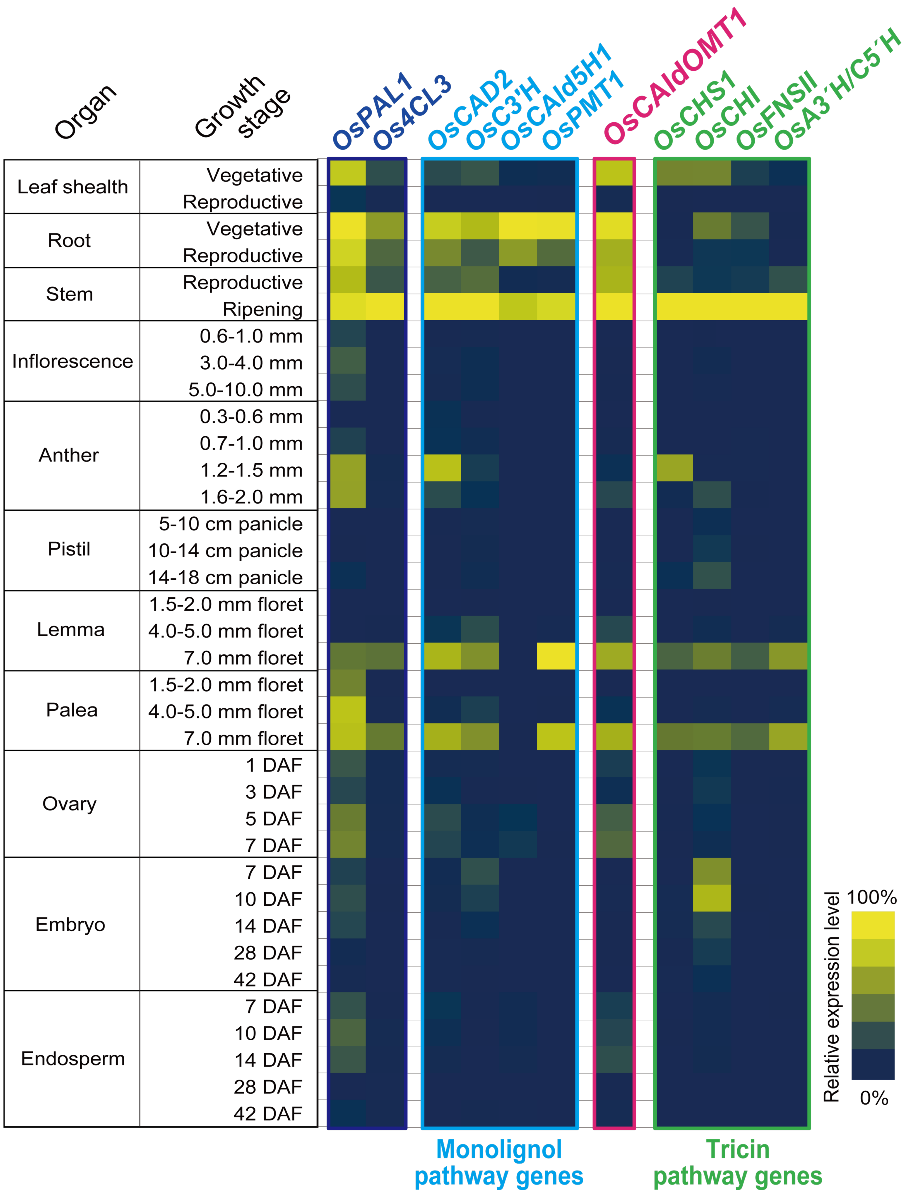

Figure S2. *In silico* gene expression analysis of monolignol and tricin biosynthetic genes in wild-type rice. Microarray data were obtained from Rice Expression Profile Database (Sato *et al.*, 2012). DAF: days after flowering.

Accession numbers of the genes used for *in silico* expression analysis can be found in the EMBL/GenBank data libraries under accession numbers AK102817 (*OsPAL4*) (Cass *et al.*, 2015), AK070083 (*Os4CL3*) (Gui *et al.*, 2011), AK105011 (*OsCAD2*) (Koshiba *et al.*, 2013b), AK099695 (*OsC3'H*) (Takeda *et al.*, 2018), AK067847 (*OsCAld5H1*) (Takeda *et al.*, 2017, Takeda *et al.*, 2019), AK060689 (*OsPMT1*) (Withers *et al.*, 2012), AB00801 (*OsCHS1*) (Shih *et al.*, 2008), AK061390 (*OsCHI*) (Shih *et al.*, 2008, Hong *et al.*, 2012), AK100972 (*OsFNSII*) (Lam *et al.*, 2014, Lam *et al.*, 2017), and AK070442 (*OsA3'H/C5'H*) (Lam *et al.*, 2015; Lam *et al.*, 2019).

(Figure S3)

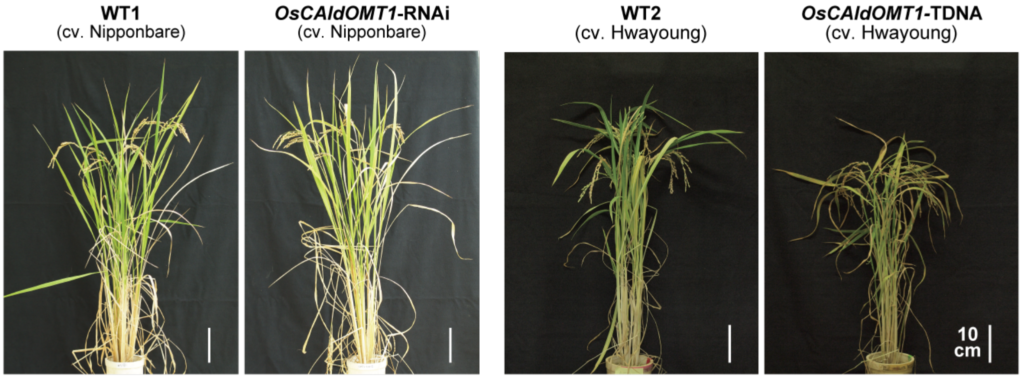

Figure S3. Morphological phenotype at the ripening stage of *OsCAldOMT1*-RNAi, *OsCAldOMT1*-TDNA and their wild-type controls (WT1 and WT2).

(Figure S4)

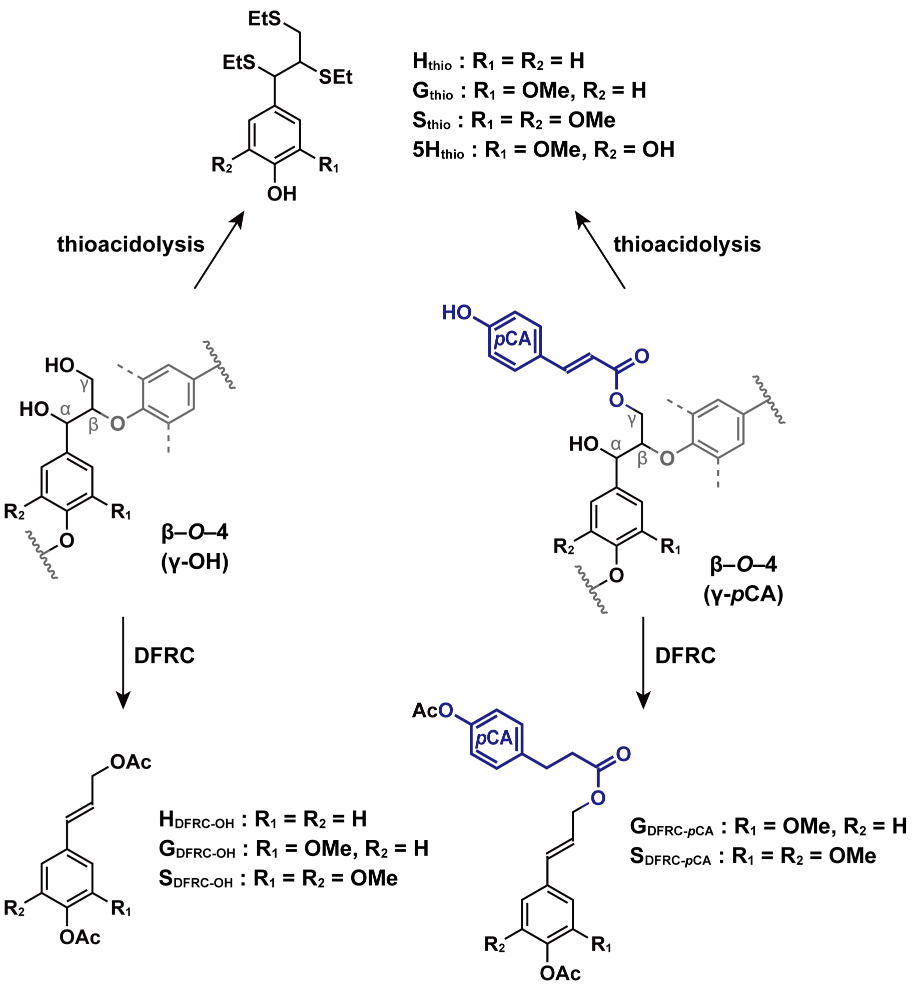

Figure S4. Lignin-derived monomeric products released by thioacidolysis and DFRC.

Both thioacidolysis and DFRC cleave β–*O*–4 linkages in the lignin polymer and release monomeric products. Unlike thioacidolysis, DFRC cleaves β–*O*–4 linkages with retaining γ-ester linkages and releases quantifiable γ-*p*-coumaroylated products from their corresponding lignin units, along with non-γ-*p*-coumaroylated products from non-acylated (γ-free) or natively acetylated (γ-OAc) units.

(Figure S5)

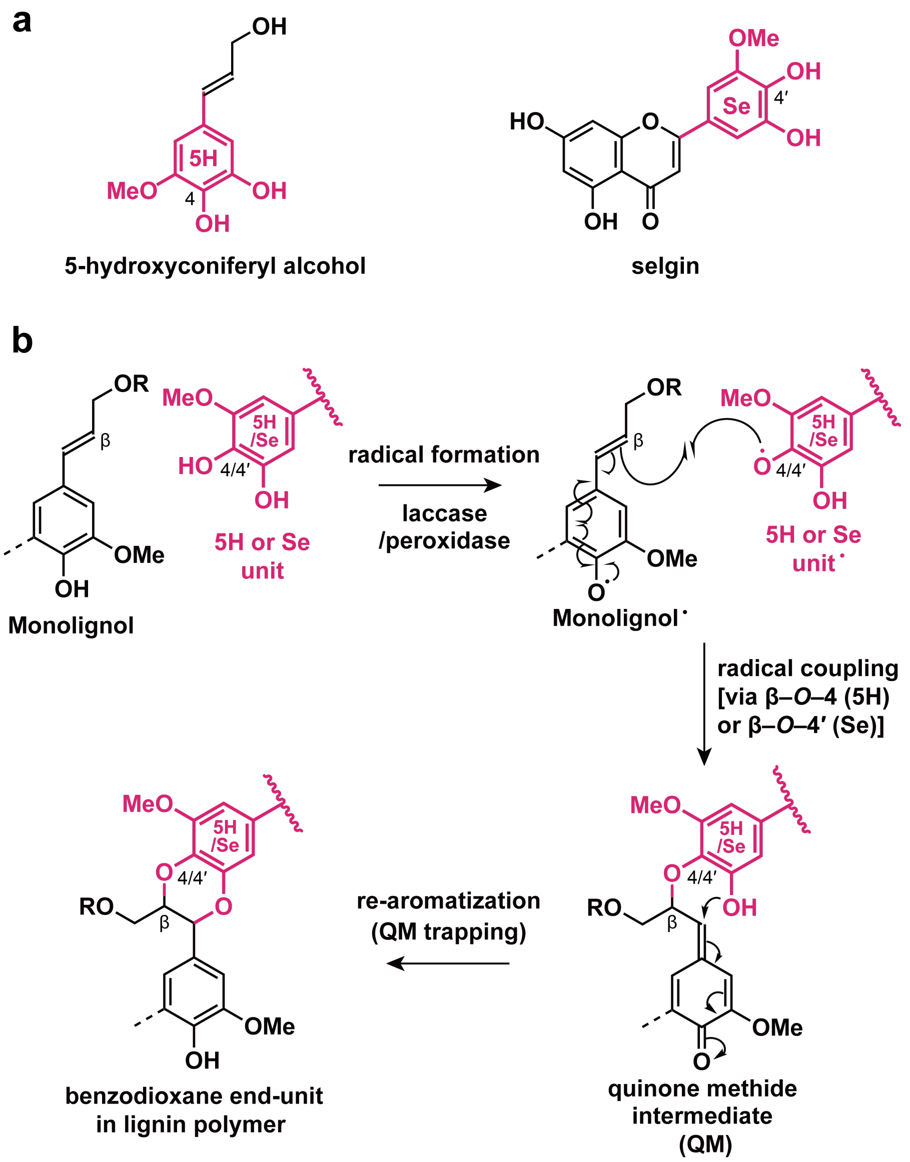

Figure S5. Non-canonical 5-hydroxyconiferyl alcohol and selgin lignin monomers (**a**) which may potentially get incorporated into lignins in *OsCAldOMT1*-deficient rice plants, and produce benzodioxane lignin units through β–*O*–4-type radical coupling followed by internal trapping of quinone methide intermediates by the *o*-hydroxyl group (**b**).

SUPPLEMENTARY REFERENCES

Cass, C. L. *et al.* Effects of *PHENYLALANINE AMMONIA LYASE* (*PAL*) knockdown on cell wall composition, biomass digestibility, and biotic and abiotic stress responses in *Brachypodium*. *J. Exp. Bot.* **66,** 4317–4335 (2015).

Daly, P. *et al.* RNAi-suppression of barley caffeic acid *O*-methyltransferase modifies lignin despite redundancy in the gene family. *Plant Biotechnol. J.* **17,** 594–607 (2019).

Eudes, A. *et al.* SbCOMT (Bmr12) is involved in the biosynthesis of tricin-lignin in sorghum. *PloS One* **12,** e0178160 (2017).

Fornalé, S. *et al.* Changes in cell wall polymers and degradability in maize mutants lacking 3'-And 5'-*O*-methyltransferases involved in lignin biosynthesis. *Plant Cell Physiol.* **58,** 240–255 (2016).

Fu, C. *et al.* Genetic manipulation of lignin reduces recalcitrance and improves ethanol production from switchgrass. *Proc. Natl. Acad. Sci. U.S.A.* **108,** 3803–3808 (2011).

Goujon, T. *et al.* A new *Arabidopsis thaliana* mutant deficient in the expression of *O*-methyltransferase impacts lignins and sinapoyl esters. *Plant Mol. Biol.* **51,** 973–989 (2003).

Gui, J., Shen, J. & Li, L. Functional characterization of evolutionarily divergent 4-coumarate: coenzyme A ligases in rice. *Plant Physiol.* **157,** 574–586 (2011).

Guo, D., Chen, F., Inoue, K., Blount, J. W. & Dixon, R. A. Downregulation of caffeic acid 3-*O*-methyltransferase and caffeoyl CoA 3-*O*-methyltransferase in transgenic alfalfa: impacts on lignin structure and implications for the biosynthesis of G and S lignin. *Plant Cell.* **13,** 73–88 (2001).

Ho-Yue-Kuang, S. et al. Mutation in *Brachypodium* caffeic acid *O*-methyltransferase 6 alters stem and grain lignins and improves straw saccharification without deteriorating grain quality. *J. Exp. Bot.* **67,** 227–237 (2015).

Hong, L. *et al.* A mutation in the rice chalcone isomerase gene causes the *golden hull and internode 1* phenotype. *Planta* **236,** 141–151 (2012).

Jung, J. H., Fouad, W. M., Vermerris, W., Gallo, M. & Altpeter, F. RNAi suppression of lignin biosynthesis in sugarcane reduces recalcitrance for biofuel production from lignocellulosic biomass. *Plant Biotechnol. J.* **10,** 1067–1076 (2012).

Koshiba, T. *et al.* Characterization of 5-hydroxyconiferaldehyde *O*-methyltransferase in *Oryza sativa*. *Plant Biotechnol.* **30,** 157–167 (2013a).

Koshiba, T. *et al.* *CAD2* deficiency causes both brown midrib and gold hull and internode phenotypes in *Oryza sativa* L. cv. Nipponbare. *Plant Biotechnol.* **30,** 365–373 (2013b).

Lam, P. Y., Liu, H. & Lo, C. Completion of tricin biosynthesis pathway in rice: cytochrome P450 75B4 is a novel chrysoeriol 5'-hydroxylase. *Plant Physiol.* **175,** 1527–1536 (2015).

Lam, P. Y. *et al.* Recruitment of specific flavonoid B-ring hydroxylases for two independent biosynthesis pathways of flavone-derived metabolites in grasses. *New Phytol.* **223**, 204–219 (2019).

Lam, P. Y. *et al.* Disrupting flavone synthase II alters lignin and improves biomass digestibility. *Plant Physiol.* **174,** 972–985 (2017).

Lam, P. Y., Zhu, F. Y., Chan, W. L., Liu, H. & Lo, C. Cytochrome P450 93G1 is a flavone synthase II that channels flavanones to the biosynthesis of tricin *O*-linked conjugates in rice. *Plant Physiol.* **165,** 1315–1327 (2014).

Ma, Q. H. & Xu, Y. Characterization of a caffeic acid 3-*O*-methyltransferase from wheat and its function in lignin biosynthesis. *Biochimie* **90,** 515–524 (2008).

Nakatsubo, T. *et al.* Roles of 5-hydroxyconiferylaldehyde and caffeoyl CoA *O*-methyltransferases in monolignol biosynthesis in *Carthamus tinctorius*. *Cell. Chem. Technol.* **41,** 511–520 (2007).

Nakatsubo, T. *et al.* A new *O*-methyltransferase for monolignol synthesis in *Carthamus tinctorius*. *Plant Biotechnol.* **31,** 545–553 (2014).

Osakabe, K. *et al.* Coniferyl aldehyde 5-hydroxylation and methylation direct syringyl lignin biosynthesis in angiosperms. *Proc. Natl. Acad. Sci. U.S.A.* **96,** 8955–8960 (1999).

Pinçon, G. *et al.* Repression of *O*-methyltransferase genes in transgenic tobacco affects lignin synthesis and plant growth. *Phytochemistry* **57,** 1167–1176 (2001).

Sato, Y. *et al.* RiceXPro version 3.0: expanding the informatics resource for rice transcriptome. *Nucleic Acids Res.* **41,** D1206–1213 (2012).

Shih, C. H. *et al.* Functional characterization of key structural genes in rice flavonoid biosynthesis. *Planta* **228,** 1043–1054 (2008).

Takeda, Y. *et al.* Regulation of *CONIFERALDEHYDE 5-HYDROXYLASE* expression to modulate cell wall lignin structure in rice. *Planta* **246,** 337–349 (2017).

Takeda, Y. *et al.* Lignin characterization of rice *CONIFERALDEHYDE 5-HYDROXYLASE* loss-of-function mutants generated with the CRISPR/Cas9 system. *Plant J.* **97,** 543–554 (2019).

Takeda, Y. *et al.* Downregulation of p-*COUMAROYL ESTER 3-HYDROXYLASE* in rice leads to altered cell wall structures and improves biomass saccharification. *Plant J.* **95,** 796–811 (2018).

Weng, J. K., Akiyama, T., Ralph, J. & Chapple, C. Independent recruitment of an *O*-methyltransferase for syringyl lignin biosynthesis in *Selaginella moellendorffii*. *Plant Cell* **23,** 2708–2724 (2011).

Withers, S. *et al.* Identification of grass-specific enzyme that acylates monolignols with *p*-coumarate. *J. Biol. Chem.* **287,** 8347–8355 (2012).

Zhang, K. *et al.* *GOLD HULL AND INTERNODE2* encodes a primarily multifunctional cinnamyl-alcohol dehydrogenase in rice. *Plant Physiol.* **140**, 972–983 (2006).
